# TRPM2 regulates TXNIP-mediated NLRP3 inflammasome activation via interaction with p47 phox under high glucose in human monocytic cells

**DOI:** 10.1038/srep35016

**Published:** 2016-10-12

**Authors:** Hisa Hui Ling Tseng, Chi Teng Vong, Yiu Wa Kwan, Simon Ming-Yuen Lee, Maggie Pui Man Hoi

**Affiliations:** 1 State Key Laboratory of Quality Research in Chinese Medicine, Institute of Chinese Medical Sciences, University of Macau, Avenida da Universidade, Taipa, Macau, China; 2 School of Biomedical Sciences, Faculty of Medicine, The Chinese University of Hong Kong, Shatin, N.T., Hong Kong, China

## Abstract

Excessive production of reactive oxygen species (ROS) induced by hyperglycemia increased the secretion of interleukin-1β (IL-1β), which contributes to the pathogenesis of diabetes and its complications. Although high glucose (HG)-induced oxidative stress and aberrant Ca^2+^ channels activity causes an increase in transmembrane Ca^2+^ influx, however the relative contribution of Transient receptor potential (TRP) channels is not well studied. Here, we identified that HG (30 mM glucose for 48 h) induced the activation of the NLRP3-ASC inflammasome, leading to caspase-1 activation, and IL-1β and IL-18 secretion in human monocytic cell lines. Moreover, we used a hyperglycemia model in U937 monocytes, showing that the activation of TRPM2 was augmented, and TRPM2-mediated Ca^2+^ influx was critical for NLRP3 inflammasome activation. This pathway involved NADPH oxidase-dependent ROS production and TXNIP-NLRP3 inflammasome pathway. Furthermore, the inhibition of TRPM2 reduced ROS production and lowered NADPH oxidase activity via cooperatively interaction with p47 phox in response to HG. These results provided a mechanistic linking between TRPM2-mediated Ca^2+^ influx and p47 phox signaling to induce excess ROS production and TXNIP-mediated NLRP3 inflammasome activation under HG, and suggested that TRPM2 represented a potential target for alleviating NLRP3 inflammasome activation related to hyperglycemia-induced oxidative stress in Type 2 diabetes Mellitus (T2DM).

Type 1 and type 2 diabetes Mellitus (T2DM) are associated with chronic low-grade inflammation, with pro-inflammatory profile of the activated monocytes that contribute to the progression to diabetic complication^1^,^2^,^3^. Interleukin 1β (IL-1β) is a key pro-inflammatory cytokine involved in type 1 diabetes and T2DM, and participate in the pathogenesis of atherosclerosis^2^,^4^. Indeed, IL-1β has been implicated in a variety of metabolic diseases, which are associated with endogenous metabolic danger signals that trigger NLRP3 inflammasome activation and contribute to chronic inflammation^5^,^6^. NLRP3 inflammasome is a multiprotein complex, which mediates the activation of caspase-1 and IL-1β maturation, and IL-1β secretion was known to contribute to β-cells failure and insulin resistance in T2DM^7^,^8^. Recently, several studies suggested that high glucose (HG), a characteristic of T2DM, acts as a metabolic trigger of NLRP3 inflammasome in various cell types, including cardiomyocytes, adipose tissue and pancreatic islet cells^8^,^9^,^10^, therefore this confirmed the importance of NLRP3 inflammasome in the development of T2DM.

Thioredoxin-interacting protein (TXNIP) was identified as a binding partner of NLRP3 inflammasome, and participated in ROS-dependent NLRP3 inflammasome activation after its dissociation with thioredoxin (Trx)^11^. TXNIP has been shown to mediate HG-induced activation of NLRP3 inflammasome by ROS signaling pathway in mesangial cells^12^. The TXNIP expression was reported to be upregulated by HG in β-cells, mesangial cells and adipose tissue^8^,^12^,^13^. Importantly, TXNIP could also regulate HG-induced ROS production, which required mitochondria and NADPH oxidase^14^, hence it might play a crucial role in controlling ROS production and its downstream signaling.

Recently it has been shown that HG induced oxidative stress-mediated Ca^2+^ influx via Transient receptor potential (TRP) channels in human monocytes^11^. It has been recognized that Transient receptor potential melastatin-2 (TRPM2) acts as a sensor for ROS, and was linked to the regulation of ROS production and ROS-mediated inflammation in human monocytes/macrophages^15^,^16^. TRPM2 channel is a Ca^2+^ permeable, non-selective cation channel, which expresses abundantly in immune cells, and it contains an intracellular enzymatic domain that possesses ADP-ribose (ADPR) hydrolase activity^17^,^18^. ADPR was primarily generated by poly (ADPR) polymerase 1 (PARP-1), which is a nuclear enzyme that participates in oxidative stress and DNA damage^19^. Thus, we hypothesized that TRPM2 might link oxidative stress to ROS-mediated NLRP3 inflammasome activation under HG condition in human monocytes.

Our study was to investigate the functional role of TRPM2 under hyperglycemic environment in human monocytic cell lines. We demonstrated that HG induced NADPH oxidase-dependent ROS production, which subsequently triggered Ca^2+^ influx via TRPM2. Moreover, we showed that HG-mediated Ca^2+^ influx via TRPM2 caused p47 phox activation, which contributed to ROS production and TXNIP-mediated NLRP3 inflammasome activation. Therefore, our results suggested a novel mechanism of TRPM2-p47 phox in regulating HG-induced NLRP3 inflammasome activation in human monocytes.

## Results

### HG upregulated TRPM2 expression and enhanced its activity via ROS production in U937 cells

**Figure 1:**
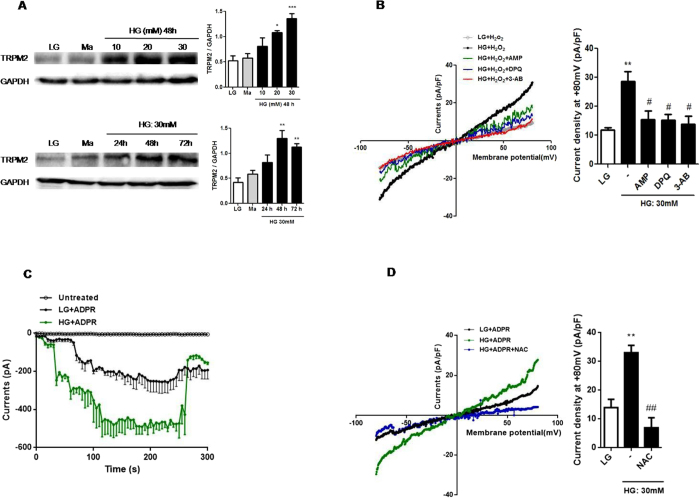
HG enhanced TRPM2 protein expression and its activity via ROS production in U937 monocytes. (**A**) Representative immunoblots and graphs for TRPM2 expression induced by low glucose (LG; 5.5 mM glucose), mannitol (Ma; 30 mM), high glucose (HG; 10, 20, 30 mM glucose for 48 h, or 30 mM glucose for 24, 48, 72 h) in U937 cells; Relative expression of TRPM2 protein was normalized to GAPDH (n = 4). (**B–D**) Representative current-voltage relationship and TRPM2-like current density induced at +80 mV (**B**) by extracellular H_2_O_2_ (1  mM) in the presence of adenosine monophosphate (AMP; 100 μM), or pre-treatment with 3-aminobenzamide (3-AB; 5 mM) or 3,4-dihydro-5-[4-(1-piperidinyl)butoxy]-1(2H)-isoquinolinone (DPQ; 100 μM) (n = 6–8) or (**C**) by intracellular denosine 5′-diphosphoribose (ADPR; 1 mM) under LG or HG (30 mM glucose for 48 h) in U937 cells (n = 7–10), or (**D**) by intracellular ADPR (1 mM) with pre-treatment of N-acetyl-L-cysteine (NAC; 25 mM) under HG condition in U937 cells (n = 6–9). Data shown were representative of four or five independent experiments (mean ± S.E.M.). (**A,B**) *P < *0.05*, **P < *0.01* and ***P < *0.001* vs. LG; ^*#*^*P* < *0.05* vs. HG.

It is generally accepted that TRPM2 is activated by cellular oxidative stress, such as H_2_O_2_^18^. Long-term hyperglycemia environment-induced oxidative stress contributed to the pathogenesis and progression of T2DM^20^, however the role of TRPM2 under HG condition is still unknown. To determine whether hyperglycemia altered TRPM2 function, we first treated U937 monocytes with various glucose concentrations for different timepoints. We observed that TRPM2 expression was increased in dose- (10, 20 and 30 mM) and time-dependent (24, 48 and 72 h) manners, but it was not affected by 5.5 mM glucose (Low glucose; LG) or 30 mM mannitol (Ma; osmotic control) (Fig. 1A). Thus, treatment with 30 mM glucose for 48 h was regarded as HG. H_2_O_2_ was proposed to gate TRPM2 channel directly in immune cells, such as macrophage or monocytes^16^,^21^. We also demonstrated that HG stimulation amplified intracellular H_2_O_2_-evoked TRPM2-like currents in U937 cells by whole-cell patch clamp, whereas these effects were attenuated by DPQ or 3-AB (PARP inhibitors, to abolish ADPR generation), as well as AMP (a potent TRPM2 inhibitor^22^) (Fig. 1B). Consistently, intracellular perfusion with ADPR (a TRPM2 intracellular agonist) also evoked cationic currents, and these currents were also markedly enhanced under HG condition (Fig. 1C,D). Meanwhile, we further observed that pre-treatment with NAC (a ROS inhibitor) could also markedly inhibited HG-enhanced ADPR-evoked TRPM2-like currents (Fig. 1D), indicating that ROS production was essential for the sensitization of TRPM2 to HG in human monocytes.

### TRPM2 regulated HG-induced NLRP3-dependent IL-1β secretion in human monocytic cells

**Figure 2:**
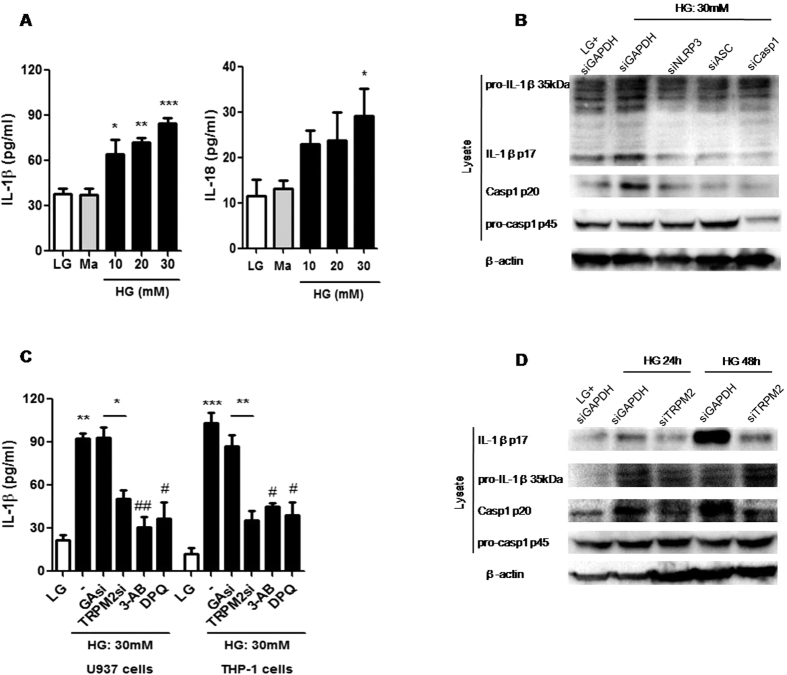
TRPM2 was required for HG-induced caspase-1-dependent IL-1β secretion in U937 monocytes. (**A,C**) ELISA for IL-1β or IL-18 secretion from the supernatants of treated cells. (**A**) U937 cells were stimulated with low glucose (LG; 5.5 mM glucose), mannitol (Ma; 30 mM), high glucose (HG; 10, 20, 30 mM glucose) for 48 h (n = 6–7). (**B,D**) Representative immunoblots for pro-IL-1β, IL-1β p17, pro-caspase-1, cleaved caspase-1 (p20), GAPDH and β-actin in the presence of (**B**) GAPDH-, NLRP3-, ASC- or caspase-1-siRNA, or (**D**) GAPDH- or TRPM2-siRNAs under HG. (**C**) U937 or THP-1 cells were stimulated with HG in the presence of GAPDH- or TRPM2-siRNAs, or 3-AB (5 mM) or DPQ (100 μM) (n = 4). Data shown were shown as mean ± S.E.M. (**A,C**) *P < *0.05*, **P < *0.01* and ***P < *0.001* vs. LG; ^*#*^*P* < *0.05* and ^*##*^*P* < *0.01* vs. HG.

It has been reported that HG induced IL-1β secretion in human monocytes^23^. Here, we observed that HG, but not Ma, significantly induced IL-1β or IL-18 secretion in a dose-dependent manner (Fig. 2A), and this effect was significantly suppressed by pan-caspase inhibitor, zVAD, or caspase-1-specific inhibitor, zYVAD (Supplementary Fig. S1A) in U937 monocytes. Western blot results showed the knockdown efficiencies of NLRP3, ASC and caspase-1 by specific small interfering RNA (siRNA) in U937 cells (Supplementary Fig. S1B), then we further examined the involvement of NLRP3 inflammasome in IL-1β secretion in response to HG. Our results showed that HG induced the maturation of IL-1β (p17), cleavage of caspase-1 (p20) and IL-1β secretion, but not in NLRP3-, ASC- or caspase-1-siRNA-treated U937 cells (Fig. 2B and Supplementary Fig. S1C). To determine whether NLRP3 inflammasome activation was specific for HG-mediated IL-1β secretion, the effect of HG-induced IL-1β secretion on NLRC4 inflammasome activation was assessed. However, HG-induced IL-1β secretion remained unchanged in NLRC4-siRNA-treated cells (Supplementary Fig. S1C). Similar effects were obtained in THP-1 human monocytic cells (Supplementary Fig. S1D), further confirmed a role of NLRP3 inflammasome in HG-induced caspase-1-dependent IL-1β secretion in human monocytic cells. In addition, we demonstrated that HG-induced IL-1β secretion was inhibited by TRPM2-siRNA or TRPM2 inhibitors, 3-AB and DPQ, in U937 and THP-1 cells (Fig. 2C). The knockdown efficiency of TRPM2 was shown in Supplementary Fig. S1B. Similar results were obtained by HG on the maturation of IL-1β (p17) and cleavage of caspase-1 (p20) (Fig. 2D and Supplementary Fig. S1E). Unlike IL-1β secretion, HG-induced TNF-α and IL-6 secretions were unaffected by TRPM2-siRNA-treated U937 cells (Supplementary Fig. S1F). Thus, these results indicated that TRPM2 was required for HG induced the activation of NLRP3 inflammasome, which mediated the secretions of IL-1β and IL-18 in human monocytes.

### HG-evoked Ca^2+^ influx via TRPM2 was necessary for NLRP3 inflammasome activation in U937 cells

**Figure 3:**
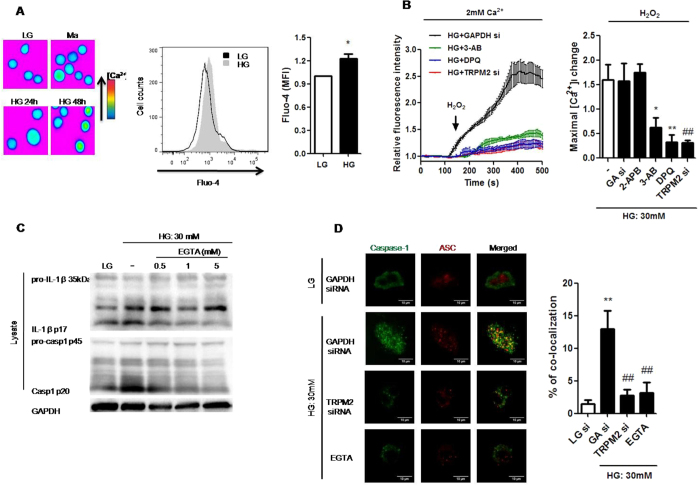
Ca^2+^ influx via TRPM2 was required for HG-induced NLRP3 inflammasome activation in U937 cells. (**A**) Fluorescence images showing fluo-4-loaded cells taken at indicated timepoints, and representative flow cytometric plot and graph showing relative median fluorescence intensity (MFI) of intracellular Ca^2+^ concentration by Fluo-4 staining under low glucose (LG; 5.5 mM glucose;) or high glucose (HG; 30 mM glucose) (n = 5). (**B**) Relative changes in [Ca^2+^]_i_, evoked by H_2_ O_2_ (1 mM) over the time course. The cells were treated in the presence of 2-aminoethoxydiphenyl borate (2-APB; 100 μM), or with pre-treatment of 3-aminobenzamide (3-AB; 5 mM) or 3,4-dihydro-5-[4-(1-piperidinyl)butoxy]-1(2H)-isoquinolinone (DPQ; 100 μM), or GAPDH- (GA si) or TRPM2-siRNAs (TRPM2 si) under HG (n = 4). (**C**) Representative immunoblots for pro-IL-1β, IL-1β p17, pro-caspase-1, cleaved caspase-1 (p20), and GAPDH in the presence of EGTA-AM (0.5, 1, 5 mM) under HG (n = 4). (**D**) Immunofluorescence images showing the location of caspase-1 and ASC in fixed cells using confocal microscopy in the presence of GAPDH- or TRPM2-siRNAs, or EGTA-AM (5 mM) under HG. The percentage of co-localization of caspase-1 with ASC was calculated as the average volume of the overlapping areas (n = 4). Data were shown as mean ± S.E.M. (**A**) *P < *0.05* vs. LG. (**B**) *P < *0.05* and **P < *0.01* vs. HG; ^*##*^*P* < *0.01* vs. HG + GA si. (**D**) **P < *0.01* vs. LG + GA si; ^*##*^*P* < *0.01* vs. HG + GA si.

Previous studies suggested that HG increased transmembrane Ca^2+^ influx in human monocytes^11^. Consistently, we observed an increase in intracellular Ca^2+^ level under HG in U937 cells (Fig. 3A). Moreover, we also demonstrated that H_2_O_2_-evoked Ca^2+^ influx was enhanced under HG condition (Supplementary Fig. S2A), whereas this enhancement was blocked by TRPM2-siRNA, DPQ or 3-AB but not by 2-APB, a blocker of store-operated Ca^2+^ entry (Fig. 3B), suggesting HG-induced intracellular Ca^2+^ increase was partially through TRPM2-mediated Ca^2+^ influx. Since Ca^2+^ signaling was reported to participate in NLRP3 inflammasome activation^24^, therefore we examined whether Ca^2+^ signaling participated in regulating NLRP3 inflammasome activation under HG. In U937 cells, HG-induced IL-1β secretion, IL-1β (p17) maturation, and caspase-1 (p20) cleavage were inhibited by blocking extracellular Ca^2+^ with EGTA (Fig. 3C and Supplementary Fig. S2B). Consistent with this, HG-induced IL-1β secretion was also suppressed by BAPTA-AM (an intracellular Ca^2+^ chelator) (Supplementary Fig. S2B). Similar results with EGTA or BAPTA-AM were also shown in THP-1 cells (Supplementary Fig. S2B), suggesting that HG could elevate intracellular Ca^2+^ to induce IL-1β secretion. Furthermore, confocal microscopy results confirmed that HG induced ASC/caspase-1 co-localization compared to LG, whereas this co-localization was reduced by TRPM2-siRNA or EGTA treatment (Fig. 3D). Therefore, these results strongly suggested that TRPM2-mediated Ca^2+^ influx was involved in NLRP3 inflammasome activation under HG condition.

### TRPM2-mediated Ca^2+^ influx was required for p47 phox phosphorylation and TXNIP/Trx pathway under HG in U937 cells

**Figure 4:**
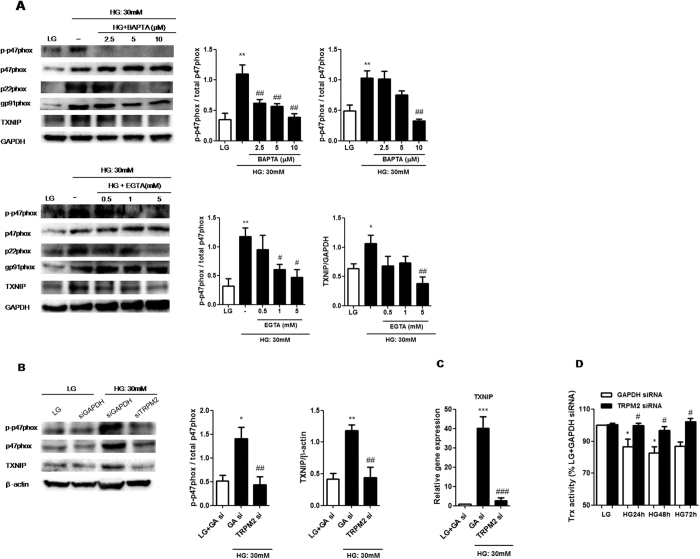
TRPM2 knockdwon markedly reduced p47 phox phosphorylation, TXNIP expression and Trx activity under HG in U937 monocytes. (**A**) Representative immunoblots and graphs for protein expressions of p47 phox, p22phox, gp91pox, TXNIP or GAPDH in the presence of BAPTA-AM (2.5, 5, 10 μM), or EGTA (0.5, 1, 5 mM) under low glucose (LG; 5.5 mM glucose) or high glucose (HG; 30 mM glucose) (n = 4–5). (**B**) Representative immunoblots and graphs for protein expressions of p47 phox, TXNIP or GAPDH or β-actin in the presence of GAPDH- or TRPM2-siRNA under HG (n = 4). (**C**) Quantitative PCR was performed on TXNIP mRNA in GAPDH- (GA si) or TRPM2-siRNA-treated cells under LG or HG, and it was normalized to LG + GAPDH-siRNA (n = 4). (**D**) TRX activity was determined by the insulin disulfide reduction assay, and was normalized to LG + GAPDH-siRNA. The cells were stimulated with HG for 24, 48, 72 h in the presence of GAPDH- or TRPM2-siRNA (n = 4). Data were shown as mean ± S.E.M. (**C,D**) *P < *0.05* and ***P < *0.001* vs. LG + GAPDH-siRNA; ^*#*^*P* < *0.05* and ^*###*^*P* < *0.001* vs. HG + GADPH-siRNA.

HG-induced IL-1β secretion was shown to be dependent on protein kinase C-α (PKC-α)-NADPH oxidase (p47 phox) pathway in human monocytes^23^, and this pathway could be activated by intracellular Ca2+ 25,26. Therefore, we next examined whether TRPM2-mediated Ca^2+^ influx was an upstream of NADPH oxidase to regulate NLRP3 inflammasome activation. Supplementary Fig. S3A showed that HG increased the protein expressions of p47 phox (an essential component of NADPH oxidase) and another NADPH oxidase components, p22phox in a dose-dependent manner in U937 cells. Buffering [Ca^2+^]_i_ with BAPTA-AM or removal of extracellular Ca^2+^ with EGTA inhibited HG-induced phosphorylated p47 phox and p22 phox expression, but not affecting gp91 phox expression (Fig. 4A and Supplementary Fig. S3B). In addition, we demonstrated that TRPM2-siRNA markedly attenuated HG-induced p47 phox activation, but not on p22 phox, gp91 phox expressions (Fig. 4B and Supplementary Fig. S3C). Similar results were obtained by 3-AB or DPQ (Supplementary Fig. S3D). In contrast to p47 phox activation, TRPM2-siRNA did not affect HG-induced phosphorylated PKC-α and phosphorylated ERK1/2, a downstream of PKC-α (Supplementary Fig. S3C), suggesting HG amplified Ca^2+^ influx via TRPM2 to activated p47 phox activation. Interestingly, we found that HG enhanced H_2_O_2_-evoked Ca^2+^ influx, which was suppressed by p47 phox-siRNA (Supplementary Fig. S3E), indicating that the active p47 phox might in turns amplify H_2_O_2_-evoked Ca^2+^ influx under HG condition.

HG stimulation was reported to induce oxidative stress and cytokine secretions through TXNIP signaling in human THP-1 monocytes^27^. We also confirmed that HG increased TXNIP protein and mRNA expressions in a dose-dependent manner in U937 cells (Supplementary Fig. S3A,F). Since HG-induced up-regulation of TXNIP was accompanied with p47 phox activation, so we postulated that TRPM2 was an upstream of p47 phox activation and might contribute to the up-regulation of TXNIP mRNA and protein. To examine this, we blocked Ca^2+^ signaling and knockdown TRPM2 by siRNA under HG in U937 cells. Figure 4B,C showed that the up-regulation of TXNIP protein and mRNA expressions by HG were decreased in TRPM2-siRNA-treated cells compared to LG. Moreover, the increase in TXNIP protein expression by HG was attenuated by BAPTA (Fig. 4A), EGTA (Fig. 4A), or 3-AB and DPQ (Supplementary Fig. S3D), indicating that TRPM2 was required for Ca^2+^-dependent HG-induced TXNIP upregulation. Since TXNIP acts as a negative regulator of Trx activation, an antioxidative protein^28^, next we measured Trx activity under HG condition. Figure 4D showed that HG decreased Trx activity in U937 cells, whereas TRPM2-siRNA markedly prevented this decrease, this further suggested that TRPM2 was involved in the regulation of TXNIP and Trx activity under HG condition.

### HG induced the interaction of TRPM2 with p47 phox in U937 monocytes

**Figure 5:**
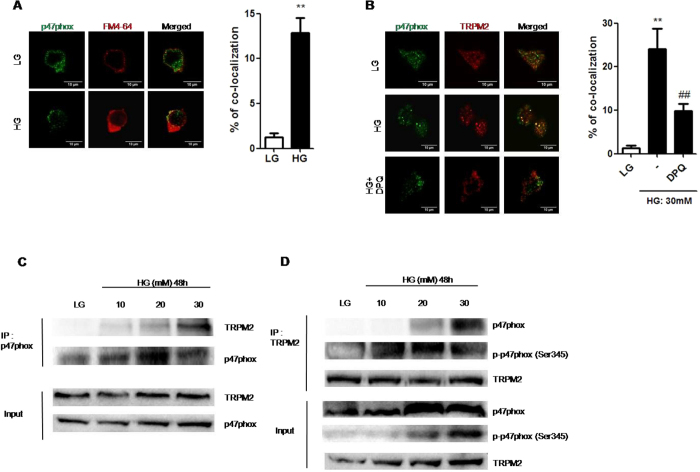
TRPM2 interacted with p47 phox during HG stimulation in U937 monocytes. (**A,B**) Immunofluorescence images showing the location of the (**A**) FM4-64 (1 uM; cell membrane marker) and subcellular p47 phox, or (**B**) subcellular p47 phox and TRPM2, in fixed cells by using confocal microscopy. The cells were pre-treated with 3,4-dihydro-5-[4-(1-piperidinyl)butoxy]-1(2H)-isoquinolinone (DPQ; 100 μM) under low glucose (LG; 5.5 mM glucose;) or high glucose (HG; 30 mM glucose). The percentage of co-localization of p47 phox with (**A**) FM4-64, or (**B**) TRPM2, was calculated as the average volume of the overlapping areas (n = 4–5). (**C,D**) Representative immunoblots showing the immunoprecipitation results for the interaction of TRPM2 with p47 phox or phosphorylated-Ser345 p47 phox under high glucose (10, 20, 30 mM glucose) for 48 h (n = 4). Data were shown as mean ± S.E.M. (**A,B**) **P < *0.01* vs. LG; ^*##*^*P* < *0.01* vs. HG.

Figure 5A showed that p47 phox was located mainly in the cytosol under LG condition. After stimulation with HG, p47 phox approached to the cell membrane, which resulted in co-localization with FM4-64, a cell membrane marker, in U937 cells (Fig. 5A). Notably, we found that HG significantly promoted p47 phox co-localization with TRPM2 in U937 cells, and this effect was prevented by DPQ (Fig. 5B). Moreover, immunoprecipitation results further confirmed that HG-induced interaction between p47 phox and TRPM2 was dose-dependent (Fig. 5C,D). In addition, HG also induced the interaction between phosphorylated p47 phox and TRPM2 (Fig. 5D), therefore this suggested a mechanistic link between TRPM2 activation, p47 phox translocation, and the assembly of NADPH oxidase during HG stimulation.

### Ca^2+^ influx via TRPM2 regulated ROS-mediated NLRP3 inflammasome activation via NADPH oxidase (p47 phox) and TXNIP under HG in U937 cells

**Figure 6:**
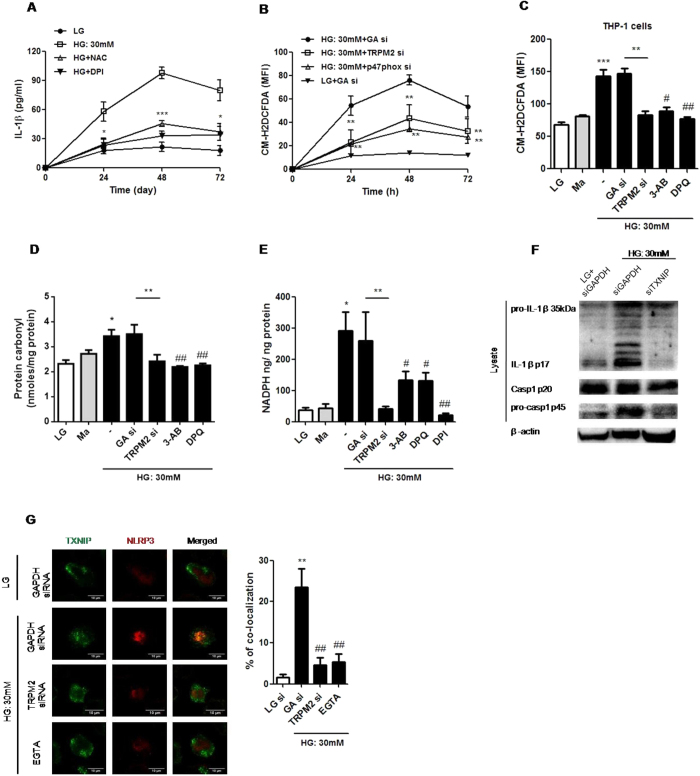
TRPM2 regulated HG-induced ROS production and NADPH oxidase activation, which linked TXNIP to NLRP3 inflammasome activation in human monocytic cell lines. (**A**) ELISA for IL-1β secretion from the supernatants of treated cells. (**B,C**) The ROS production was measured by CM-F2DCFDA staining. (**D**) The level of protein carbonyl content was measured by protein carbonyl content assay. (**E**) The cellular NADPH level was measured by NADP/NADPH assay, and NADPH oxidase activity was normalized to total cellular protein levels. (**A,B**) U937 cells were stimulated with low glucose (LG; 5.5 mM glucose) or high glucose (HG; 30 mM glucose for 24, 48, 72 h) (**A**) with pre-treatment of N-acetyl-L-cysteine (NAC; 25 mM) or diphenyleneiodonium chloride (DPI; 10 μM), or (**B**) in the presence of GAPDH- (GA si), p47 phox- or TRPM2-siRNA (n = 6–7). (**C–E**) The cells were pre-treated with 3-aminobenzamide (3-AB; 5 mM), 3,4-dihydro-5-[4-(1-piperidinyl)butoxy]-1(2H)-isoquinolinone (DPQ; 100 μM), DPI (10 μM), GAPDH- or TRPM2-siRNA under LG, mannitol (Ma; 30 mM) or HG conditions in (**C**) THP-1 cells or (**D,E**) U937 cells (n = 5–6). (**F**) Representative immunoblots for pro-IL-1β, IL-1β p17, pro-caspase-1, cleaved caspase-1 (p20), and β-actin in the presence of GADPH- or TXNIP-siRNA under HG in U937 cells (n = 5). (**G**) Immunofluorescence images showing the location of the TXNIP and NLRP3 in fixed cells by using confocal microscopy, in the presence of GAPDH- or TRPM2-siRNA, or EGTA-AM (5 mM) under HG in U937 cells (n = 4). The percentage of TXNIP co-localization with NLRP3 inflammasome was calculated as the average volume of the overlapping areas. Data were shown as mean ± S.E.M. (**A**) *P < *0.05* and ***P < *0.001* vs. HG. (**B**) **P < *0.01* vs. HG + GAPDH-siRNA. (**C–E**) *P < *0.05* and ***P < *0.001* vs. LG; ^*#*^*P* < *0.05* and ^*##*^*P* < *0.01* vs. HG. (**F**) **P < *0.01* vs. LG + GAPDH-siRNA; ^*##*^*P* < *0.01* vs. HG + GAPDH-siRNA.

Our results implicated a critical role for TRPM2 in the activation of NLRP3 inflammasome and NADPH oxidase under HG. We also demonstrated that HG-induced IL-1β secretion was strongly inhibited by NAC or DPI in U937 cells (Fig. 6A), suggesting that NADPH oxidase-dependent ROS production was involved in HG-induced NLRP3 inflammasome activation. Next, we examined other previously identified key factors that participate in NLRP3 inflammasome activation under HG including endocytosis/phagocytosis, potassium (K^+^) efflux and ATP receptor (P2 × 7 R)^29^. In contrast, cytochalasin D, an actin polymerization inhibitor to block endocytosis, or in the presence of high K^+^ or P2 × 7 R-siRNA, did not affect HG-induced IL-1β secretion (Supplementary Fig. S4A), suggesting that HG-induced NLRP3 inflammasome activation only involved NADPH oxidase-dependent ROS production, but not through K^+^ efflux, phagocytosis or P2 × 7 R.

As expected, HG increased ROS production in a dose-dependent manner (Supplementary Fig. S4B), and this effect was inhibited by TRPM2-siRNA, DPQ or 3-AB, as well as by p47 phox-siRNA (Fig. 6B and Supplementary Fig. S4B,C). Similar results were obtained in THP-1 human monocytic cells (Fig. 6C). To further examine the involvement of TRPM2 in HG-induced oxidative stress, we measured the protein carbonyl content, a protein marker of oxidative damage^30^. As expected, we observed a similar pattern in the protein carbonyl content assay, where HG significantly increased the levels of protein carbonyl contents in U937 and THP-1 cells, and these effects were prevented by TRPM2-siRNA, DPQ or 3-AB (Fig. 6D and Supplementary Fig. S4D). Moreover, HG-induced NADPH oxidase activity was attenuated by TRPM2-siRNA, DPQ or 3-AB, as well as BAPTA-AM (Fig. 6E and Supplementary Fig. S4E). These observations demonstrated an involvement of Ca^2+^ influx via TPRM2 to mediate HG-induced ROS production through NADPH oxidase (p47 phox) activation in human monocytes.

As ROS-mediated TXNIP signal was essential for mediating NLRP3 inflammasome activation^9^, we next examined whether TRPM2 mediated NLRP3 activation via ROS-TXNIP pathway under HG. Figure 6F showed that TXNIP-siRNA inhibited the maturation of IL-1β (p17) and cleavage of caspase-1 (p20) under HG in U937 cells. In addition, confocal microscopy also showed that HG triggered TXNIP and NLRP3 co-localization, whereas this co-localization was abolished by TRPM2-siRNA or the removal of extracellular Ca^2+^ with EGTA (Fig. 6G). Therefore, our results suggested that TRPM2 mediated HG-induced NLRP3 inflammasome activation via ROS-TXNIP pathway.

## Discussion

The present study aimed to investigate the role of TRPM2 channel and its physiological contribution in oxidative stress and IL-1β secretion under HG condition in U937 human monocytes. Here we showed that HG augmented TRPM2 activation to increase ROS production via interaction with p47 phox. In addition, we observed that TRPM2-dependent Ca^2+^ influx directly mediated HG-induced redox-dependent NLRP3 inflammasome activation via TXNIP in human monocytes.

Hyperglycemia contributing to inflammatory process was considered as a major risk factor for the development of T2DM and cardiovascular complications^31^,^32^. Evidences have reported that HG-induced oxidative stress elevated transmembrane Ca^2+^ influx via TRP channels in human monocytes^11^,^33^, however their relative contributions under diabetic condition have not been further studied. TRPM2 is a first identified ROS-sensitive TRP channel, and widely expressed in immune cells^34^,^35^. The function of TRPM2 was recognized as an oxidative stress and metabolic sensor, which contributed to regulate innate immunity^36^. In this study, we used a hyperglycemia model in U937 monocytes, and identified that TRPM2 could serve as a glucose sensor. ADPR was a primary gating molecule of TRPM2^17^,^37^. Activation of PARP was one of the sources of ADPR generation^36^, and it has been suggested to be occured during oxidative stress in activated monocytes^38^,^39^. Our observations further demonstrated that the inhibition of PARP attenuated the enhancement of ROS-mediated TRPM2 activation by HG, indicating that HG induced oxidative stress-mediated TRPM2 hyperactivity involving PARP. Therefore, we suggested that TRPM2 activation was augmented under HG condition as a consequence of oxidative stress in human monocytes.

Recently, TRPM2 was identified as a key factor that linked oxidative stress to NLRP3 inflammasome^40^, thus we examined whether TRPM2 could mediate HG-induced NLRP3 inflammasome activation in human monocytes. Although the activation of NLRP3 inflammasome under HG is unknown in human monocytes, its role in various cell types, including cardiomyocytes, adipose tissue and pancreatic islet cells has been proposed^8^,^9^,^10^, which linked the pathogenesis of T2DM and its complication to chronic inflammation^7^,^41^. HG could induce IL-1β secretion in human monocytes^8^,^42^, and we further demonstrated that it was dependent on NLRP3 inflammasome in U937 and THP-1 human monocytic cells. Moreover, we found that Ca^2+^ influx via TRPM2 could act as a critical regulator in HG-induced NLRP3 inflammasome activation. Ca^2+^ mobilization was necessary for triggering NLRP3 inflammasome activation by regulating its assembly and mediating ROS production and mitochondrial destabilization^24^,^43^. NLRP3 inflammasome is a protein complex that consists of an NLRP3 inflammasome sensor, the adaptor protein ASC and caspase-1^41^. Our results confirmed that Ca^2+^ influx directly regulated caspase-1/ASC inflammasome complex under HG. Moreover, we suggested that the role of TRPM2 in HG-induced NLRP3 inflammasome activation was not only mediating Ca^2+^ influx but also regulated ROS production. Although a study suggested that TRPM2 inhibited ROS production during endotoxin that caused acute inflammation in phagocytes^15^, our study and another study suggested that TRPM2 deficiency reduced oxidative stress and lowered NADPH oxidase activity during HG or after ischemia^44^, this supported the concept that TRPM2 might promote ROS production during chronic diseases. NADPH oxidase represents a major source of ROS production^45^, and was thought to contribute to HG-induced oxidative stress and IL-1β secretion through p47 phox (an organizer of NADPH oxidase)-PKC-α pathway^23^. Furthermore, oxidative stress-induced translocation of p47 phox has been reported to be regulated by [Ca^2+^]_i_ change^46^, and we further provided a strong link between Ca^2+^ influx via TRPM2 and NADPH oxidase activation, demonstrating that there was an interaction between TRPM2 and p47 phox or phosphorylated p47 phox under HG, which contributed to the activation of NLRP3 inflammasome. Therefore, we suggested a role for TRPM2-mediated Ca^2+^ mobilization in NADPH oxidase (p47 phox) assembly and its contribution to ROS production, and they act as an upstream of NLRP3 inflammasome activation in human monocytes.

TXNIP was critical for mediating NLRP3 inflammasome activation^9^, and could act as a sensor to modulate the levels of redox signaling molecules^47^. TXNIP was also reported to implicate in the pathogenesis of type 1 diabetes and T2DM. Our results demonstrated that HG triggered the binding of TXNIP to NLRP3 inflammasome in U937 monocytes, whereas Ca^2+^ influx and TRPM2 were required for this process. TXNIP has been suggested to be translocated to the cytosol upon NLRP3 inflammasome activation, where it disassociated with Trx and reduced the activity of Trx^9^. Our results also found that TRPM2 regulated the activity of Trx and NADPH oxidase under HG, indicating that TRPM2 regulated NLRP3 inflammasome activation through Trx system (consists of Trx, TXNIP, Trx reductase and NADPH^48^) under HG. Several studies demonstrated that TXNIP was upregulated under HG condition in different cell types^14^,^49^, and also participated in ROS production^14^. Indeed, the expression of TXNIP has been shown to be directly regulated by Ca^2+^ influx^49^,^50^, and our results demonstrated that blocking Ca^2+^ influx or the inhibition of TRPM2 markedly reduced HG-upregulated TXNIP expression, suggesting that the initiation of NLRP3 inflammasome activation under HG was dependent on TRPM2-mediated ROS production and its contribution to TXNIP binding to NLRP3 inflammasome. Therefore, our results indicated that HG activated ROS-dependent NLRP3 inflammasome through TRPM2-TXNIP pathway in human monocytes.

In this study, we treated human monocytic cells with HG for 48 h to investigate the underlying mechanism of NLRP3 inflammasome in the progression of T2DM. It is well known that monocytes have a pro-inflammatory profile in diabetic animal models, as well as in T2DM patients^1^,^51^, and HG could augment this pro-inflammatory effect in monocytes^52^. Recent study have shown that NLRP3 inflammasome activation was elevated in myeloid cells from T2DM patients^53^. Moreover, a study demonstrated that TRPC6 mRNA was up-regulated in monocytes from T2DM patients, whereas HG treatment could induce TRP-mediated Ca^2+^ influx through oxidative stress^11^. Hence, our *in vitro* observations are in agreement with these *in vivo* and clinical studies, and we further provide a novel mechanism for Ca^2+^ -mediated NLRP3 inflammasome activation in the pathogenesis of T2DM.

In conclusion, we demonstrated that TRPM2 was a Ca^2+^ mediator of hyperglycemia-induced ROS production and NLRP3 inflammasome activation through cooperatively interaction with p47 phox, and regulation of TXNIP in human monocytes. In addition, our results also revealed a positive feedback loop by which TRPM2 promote NADPH oxidase-dependent ROS production, which in turn to amplified TRPM2 activation under HG. Therefore, our results supported that TRPM2 represented a potential therapeutic target for ameliorating NLRP3 inflammasome activation related to hyperglycemia-induced oxidative stress in T2DM.

## Materials and Methods

### Reagents and chemicals

2-aminoethoxydiphenyl borate (2-APB), 3-aminobenzamide (3-AB), adenosine 5′-diphosphoribose (ADPR), adenosine monophosphate (AMP), ethylene glycol tetra acetic acid (EGTA), hydrogen peroxide solution (H_2_O_2_), D-mannitol, N-acetyl-L-cysteine (NAC) were purchased from Sigma-Aldrich, US. 3,4-dihydro-5-[4-(1-piperidinyl)butoxy]-1(2 H)-isoquinolinone (DPQ) and Z-VAD-FMK (zVAD) were from Santa Cruz Biotechnology. Z-YVAD-FMK (zYVAD) was from InvivoGen, US, while cytochalasin D (cyto D), diphenyleneiodonium chloride (DPI) and BAPTA-AM were from Tocris Biosciences, US. FM4-64 Dye was from Invitrogen, US. Antibodies used for immunoblotting or immunostaining were as follows: anti-rabbit caspase-1 (2225 S, Cell Signaling, US), anti-rabbit IL-1β (sc-7884, Santa Cruz Biotechnology, US), anti-rabbit TRPM2 (LS-C160232, LifeSpan BioSciences, US), anti-goat TRPM2 (LS-B4122, LifeSpan BioSciences, US), anti-rabbit NLRP3 (15101 S, Cell Signaling, US), anti-mouse ASC (LS-C175123, LifeSpan BioSciences, US), anti-rabbit PKC-α (2056 S, Cell Signaling, US), anti-rabbit PKC-α (phospho T497) (ab76016, Abcam, US), anti-rabbit ERK1/2 (9102 S, Cell Signaling, US), anti-rabbit ERK1/2 (Thr202/Tyr204) (9101 S, Cell Signaling, US), anti-goat p22 phox (sc-11712, Santa Cruz Biotechnology, US), anti-rabbit gp91-phox (sc-20782, Santa Cruz Biotechnology, US), anti-rabbit p47 phox (4312 S, Cell Signaling, US), anti-rabbit p47 phox (phospho Ser345) (PA5-37806, Pierce, US), anti-mouse TXNIP (K0204-3, MBL Life science, Japan), anti-rabbit GAPDH (2118 S, Cell Signaling, US), anti-rabbit β-Actin (4967 S, Cell Signaling, US), goat anti-rabbit IgG-HRP (7074 S, Cell signaling, US), donkey anti-goat IgG H&L (Alexa Fluor® 647) preadsorbed (ab150135, Abcam, US), anti-rabbit IgG (H + L), F(ab’)2 Fragment (Alexa Fluor® 488 Conjugate) (4412 S, Cell Signaling, US).

### Cell culture, treatments and ELISA

Human promonocytic leukemia cells, U937 cells, were purchased from ATCC (US), and THP-1 cells were purchased from InvivoGen (US). U937 and THP-1 cells were cultured in RPMI-1640 (Gibco, US) supplemented with 10% FBS, 2 mM L-glutamine, and 100 U/mL of penicillin and streptomycin. They were authenticated and tested for contamination. In HG experiments, before stimulation with HG, cells were cultured in 5.5 mM glucose of RPMI 1640 medium for 48 h, and the cells were then changed to 10, 20, or 30 mM glucose of RPMI 1640 medium for different time points or 30 mM mannitol as an osmotic control. For the experiments using chemical inhibitors, DPI, zVAD, zYVAD or NAC were pretreated for 24 h, while DPQ or 3-AB were pretreated for 45 min. AMP, EGTA or BAPTA-AM were treated in the presence of stimulation with HG. The supernatants from U937 or THP-1 cells were collected for the detection of human IL-1β, TNF-α and IL-6 by ELISA (eBioscience, US).

### Specific small interfering RNA (siRNA) Experiments

Cells were transiently transfected with TRPM2 specific small interfering RNA (siRNA) (60 nmol/L; Ambion, US), NLRP3 siRNA (100 nmol/L; Santa Cruz Biotechnology, US), ASC siRNA (100 nmol/L; Ambion, US), caspase-1 siRNA (100 nmol/L; Ambion, US) or p47 phox siRNA (80 nmol/L; Ambion, US) by using Lipofectamine® RNAiMAX Transfection reagent (Gibco, US). The protocol was synthesized according to the manufacturer’s protocol. GAPDH siRNA was used as a control (40 nmol/L; Ambion, US). Transfection efficiency was > 70% assessed by BLOCK-iT™ Alexa Fluor® Red Fluorescent Control (Ambion, US) and western blotting. Cells were transfected with siRNA for 24 h before experiments.

### Detection of intracellular ROS

Cells were incubated in the dark with 10 μM of 5-(and 6)-chloromethyl-2′,7′-dichlorodihydrofluorescein diacetate, acetyl ester (CM-H2-DCFDA; Molecular Probes, US) for 15 min at 37 °C, and the fluorescence intensity was detected by a microplate reader (SpectaMax M5, Molecular Devices, US) using excitation at 492 nm and emission at 517 nm wavelengths.

### Protein carbonyl content assay

Cells were extracted with 100 μl of lysis buffer and the lysates were derivatized with 2,4-dinitrophenylhydrazine derivatization (DNPH), and all the procedures were followed the manufacturer’s instruction. The levels of protein carbonyl content were determined using a Protein carbonyl content Assay Kit (Abcam, US), and were expressed as nmol carbonyls/mg protein.

### NADPH oxidase activity assay

Cells were extracted with 200 μl of extraction buffer following the manufacturer’s instruction. NADPH levels were measured by using a NADP/NADPH Assay Kit (Abcam, US), and were normalized to total cellular proteins.

### Trx activity assay

Trx activity was measured by the insulin disulfide reducing assay as previously described^14^. Briefly, 50 μg of protein (from cell lysates) was incubated with 40 μl of reaction mixture (400 μl of 1 mM HEPES (pH 7.45), 160 μl of 0.2 mM EDTA, 120 μl of 40 mg/ml NADPH, and 1 ml of 10 mg/ml insulin) at 37 °C for 15 min, the reaction was initiated by the addition of 5 μl of rat Trx reductase (20 μM; Sigma-Aldrich, US) or water as a negative control. The reaction was stopped by the addition of 250 μl of stopping buffer (6 M guanidine HCl, 1 mM DTNB in 0.2 M Tris–HCl, pH 8.0). The absorbance was measured at 412 nm.

### [Ca^2+^]_i_ measurements

The intracellular Ca^2+^ concentration ([Ca^2+^]_i_) was measured in single cells as previously described^54^. Cells were loaded with Fluo-4 AM (2 μM; Molecular Probes, US) in Tyrode solution containing 136.5 mM NaCl, 5.4 mM KCl, 0.53 mM MgCl_2_, 1.8 mM CaCl_2_, 0.33 mM NaH_2_PO_4_, 5.5 mM glucose and 5.5 mM HEPES (pH 7.4, adjusted with NaOH) for 30 min at 37 °C. Fluo-4 fluorescence intensity (494 nm excitation; 506 nm emission) was sampled at 5 s intervals using a Cell^R^ system (MT20, Olympus, US). To enable comparisons between cells, the maximal change in fluorescence intensity was measured before and after H_2_O_2_ (1 mM) was added.

### Electrophysiology

Electrophysiological recordings were obtained using a voltage-clamp technique. TRPM2 whole cell recordings were measured as described^15^. Cells were plated in Tyrode solution containing 136.5 mM NaCl, 5.4 mM KCl, 0.53 mM MgCl_2_, 1.8 mM CaCl_2_. 5.5 mM glucose and 5.5 mM HEPES (pH 7.4, adjusted with NaOH). TRPM2 currents were stimulated by a series of test pulses range from −80 to + 80 mV (test pulses were 200 ms in duration and delivered at 2 s intervals). The cells were clamped at a holding potential of 0 mV. The pipette solution contained 135 mM Cs-glutamate, 8 mM NaCl, 2 mM MgCl_2_, 0.5 mM CaCl_2_, 1 mM EGTA and 10 mM HEPES (pH 7.2, adjusted with CsOH), with or without ADPR (1 mM) or H_2_O_2_ (1 mM). The recordings were made by using an Axopatch-200B amplifier, Digidata-1321 interface and pClamp10.0 software (Axon Instruments Inc., US).

### Western blot analysis

The protein was extracted with ice-cold lysis buffer, and the protein concentrations of the lysates were measured by the bicinchoninic acid kit (Pierce, US). 40 μg proteins were used and separated by 10% SDS-PAGE gels, and were transferred onto the nitrocellulose membranes. Membranes were incubated with primary antibodies (IL-1β, p22 phox, and PKC-α antibody using 1/500 dilution, GAPDH antibody using 1/4000 dilution, whereas 1/1000 dilution was used in other antibodies) overnight at 4 °C, and secondary antibodies (anti-rabbit and anti-mouse with 1/1000 dilution, and anti-goat with 1/4000 dilution) for 1 hr, and blots were developed by enhanced chemiluminescence (GE Healthcare Life Sciences, UK) with an imaging system (Bio-Rad Laboratories, US). GAPDH and β-actin were used as housekeeping controls.

### Real-time PCR analysis

Total RNA was extracted using RNeasy Mini Kit (Qiagen, US), and cDNA was synthesised using High-Capacity cDNA Reverse Transcription Kit (Applied Biosystems, US). cDNA was quantified using Taqman assays by ViiA 7 Real-Time PCR System (Applied Biosystems, US). The Taqman probes (Applied Biosystems, US) used were as follows: TXNIP (Hs01006900_g1) and β-actin endogenous control (4326315E). β-actin was used as an internal control. Gene expressions were calculated using the ∆∆Ct method, and were normalised to control.

### Immunoprecipitation

Immunoprecipitation was carried out as described^55^. U937 cells were seeded in 75 cm^2^ flask (7.5 × 10^6^ cells/flask) for treatment with different concentrations of glucose for 48 h, and the cells were extracted with 300 μl of lysis buffer (Cell signaling, US) following the manufacturer’s instruction. The resulting supernatants were subjected to immunoprecipitation by incubating with anti-p47 phox antibody or anti-TRPM2 antibody (1/50 dilution) overnight at 4 °C. p47 phox or TRPM2 immune complexes were precipitated with Protein A Agarose Beads (Cell signaling, US) for 3 h at 4 °C. The beads were then washed five times with lysis buffer, and the precipitated proteins were eluted by boiling the beads in Sample buffer (500 mM Tris (pH 6.8), 10% SDS, 15% glycerin, 0.05% bromophenol blue, 5% DTT), and the eluted samples were run on a 10% SDS-PAGE gel. Western blots were carried out using anti-p47 phox, anti-phospho-Ser345-p47 phox antibody or anti-TRPM2 primary antibodies (1/1000 dilution), and secondary antibody (1/1000 dilution).

### Immunofluorescence

Cells were seeded in confocal dishes (SPL Life Sciences, Korea), and were treated with different conditions as described in results. The cells were fixed with Fixative (Science Cell, US) for 15 min. After permeabilization with Triton X-100, the cells were blocked in 1% BSA (Sigma-Aldrich, US) for 30 min, and incubated with primary antibody (1/50 dilution) overnight at 4 °C, and then secondary antibodies (1/400 dilution) for 1 h. Images were captured with Confocal microscopy (LEICA TCS SP8, Leica Microsystems, Germany) and quantitative analysis was performed with ImageJ software.

### Statistical analysis

All data were expressed as mean ± S.E.M., and were analyzed by Graphpad Prism 5.0 (GraphPad, US). Significant differences were determined by one-way ANOVA followed by a Dunnett’s test, or two-way ANOVA followed by a Bonferroni *post hoc* test. *P* < 0.05 was considered as significant. Sample size (n) represented the number of independent experiments.

## Additional Information

**How to cite this article**: Tseng, H. H. L. *et al.* TRPM2 regulates TXNIP-mediated NLRP3 inflammasome activation via interaction with p47 phox under high glucose in human monocytic cells. *Sci. Rep.* **6**, 35016; doi: 10.1038/srep35016 (2016).

## Supplementary Material

Supplementary Information
