# Supplementary material for: TRPM2 regulates TXNIP-mediated NLRP3 inflammasome activation via interaction with p47 phox under high glucose in human monocytic cells
Source: Sci Rep. 2016 Oct 12;6:35016. doi: 10.1038/srep35016 (PMC5059733; doi:10.1038/srep35016)
Supplement: Supplementary Information [file srep35016-s1.pdf]

TRPM2 regulates TXNIP-mediated NLRP3 inflammasome activation via interaction with p47phox under high glucose in human monocytic cells

Hisa Hui Ling Tseng[*], Chi Teng Vong[*], Yiu Wa Kwan[†], Simon Ming-Yuen Lee[*], Maggie Pui Man Hoi[*]

[*]*State Key Laboratory of Quality Research in Chinese Medicine, Institute of Chinese Medical Sciences, University of Macau, Avenida da Universidade, Taipa, Macau, China*

[†]*School of Biomedical Sciences, Faculty of Medicine, The Chinese University of Hong Kong, Shatin, N.T., Hong Kong, China*

[*]Corresponding author: Dr. Maggie Pui Man Hoi, Institute of Chinese Medical Sciences University of Macau, Room 7012, N22 Research Building, Avenida da Universidade, Taipa, Macau, China. Telephone: (+853) 8822-4876; Fax: (+853) 28841358. Email address, maghoi@umac.mo.

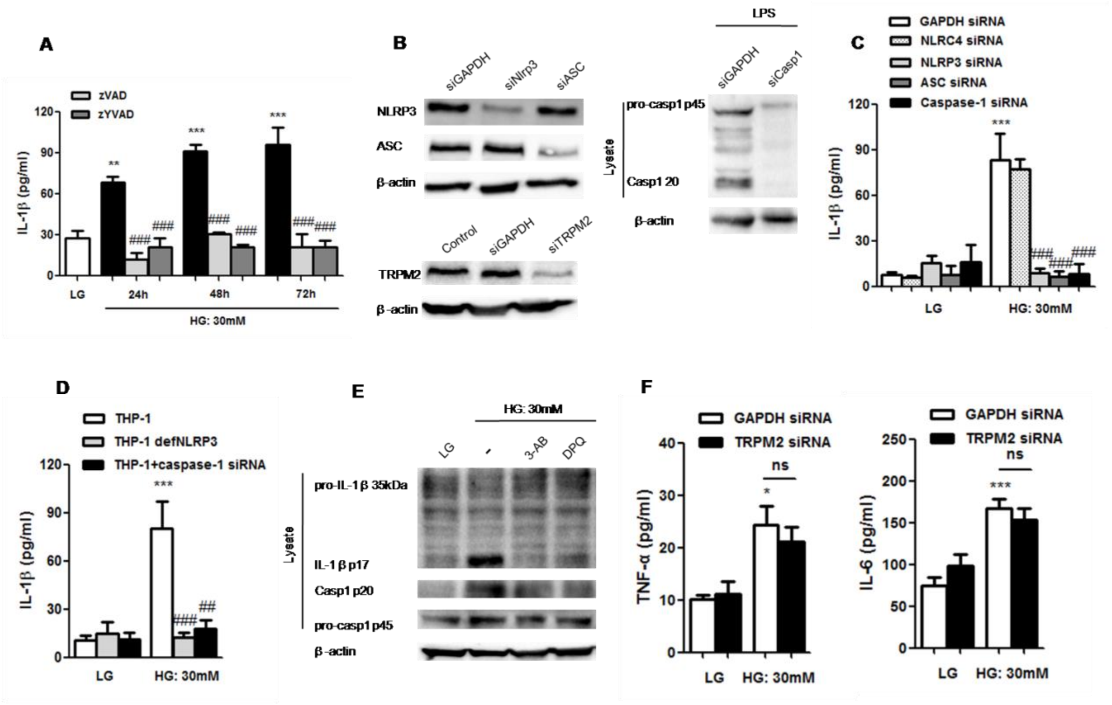

Supplementary Figure S1. (A,C,D-F) ELISA for IL-1β, TNF-α or IL-6 secretion from the supernatants of treated cells. (A) U937 cells were stimulated with low glucose (LG; 5.5mM glucose) or high glucose (HG; 30mM glucose for 24h, 48h or 72h) in the presence of Z-VAD-FMK (zVAD; 10µM) or Z-YVAD-FMK (zYVAD; 10µM) (n=5). (B) Representative immunoblots for NLRP3, ASC, TRPM2, caspase-1 and β-actin in GAPDH-, NLRP3-, ASC-, TRPM2- or caspase-1-siRNA-treated U937 cells (n=4). (C,E,F) U937 cells were stimulated with LG or HG in the presence of (C) GAPDH-, NLRP3-, ASC-, caspase-1- or NLRC4-siRNA, or (F) GAPDH- or TRPM2-siRNA (n=5). (D) THP-1 cells, and THP-1 with NLRP3 inflammasome deficient cells (THP1-defNLRP3), and caspase-1-siRNA-treated THP-1 cells were stimulated with HG (n=5). (E) Representative immunoblots for pro-IL-1β, IL-1β p17,

pro-caspase-1, cleaved caspase-1 (p20), GAPDH and β-actin in the presence of pre-treatment of 3-aminobenzamide (3-AB; 5mM) or 3,4-dihydro-5-[4-(1-piperidinyl)butoxy]-1(2H)-isoquinolinone (DPQ; 100μM) under HG (n=4). Data were shown as mean ± S.E.M. (A,D) $^{**}P<0.01$ and $^{***}P<0.001$ vs. LG; $^{\#\#}P<0.01$ and $^{\#\#\#}P<0.001$ vs. HG. (C,F) $^{*}P<0.05$ *and* $^{***}P<0.001$ vs. LG+GAPDH-siRNA; $^{\#\#\#}P<0.001$ vs. HG +GAPDH-siRNA.

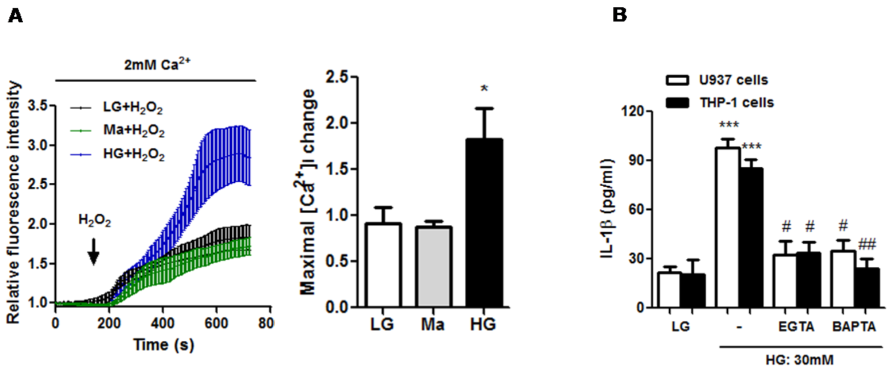

Supplementary Figure S2. (A) Relative changes in intracellular Ca^{2+} concentration ($[Ca^{2+}]_i$), evoked by H_2O_2 (1mM) over the time course. U937 cells were treated with low glucose (LG; 5.5mM glucose), mannitol (Ma; 30mM) or high glucose (HG; 30mM glucose) (n=5-6). (B) U937 cells and THP-1 cells were stimulated with LG or HG in the presence of EGTA (5mM) or BAPTA-AM (10μM) (n=5). IL-1β secretion was measured by ELISA. Data were shown as mean ± S.E.M. (A,B) $^{*}P<0.05$ and $^{***}P<0.001$ vs. LG; $^{\#}P<0.05$ and $^{\#\#}P<0.01$ vs. HG.

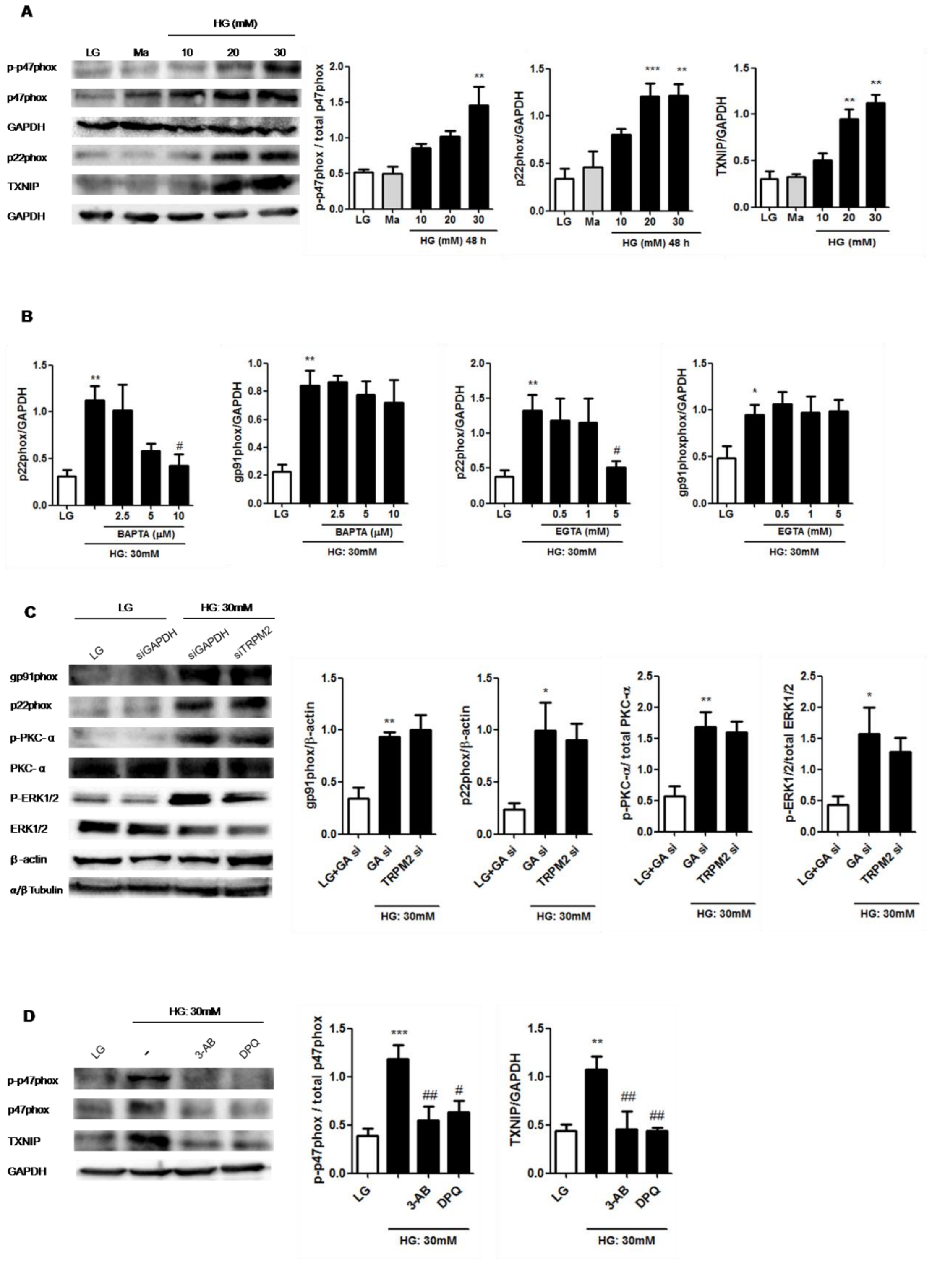
A
HG (mM)
LG
Ma
10
20
30
p-p47phox
p47phox
GAPDH
p22phox
TXNIP
GAPDH
p-p47phox / total p47phox
HG (mM) 48 h
p22phox/GAPDH
HG (mM) 48 h
TXNIP/GAPDH
HG (mM)
B
p22phox/GAPDH
BAPTA (μM)
HG: 30mM
gp91phox/GAPDH
BAPTA (μM)
HG: 30mM
p22phox/GAPDH
EGTA (mM)
HG: 30mM
gp91phoxphox/GAPDH
EGTA (mM)
HG: 30mM
C
LG
HG: 30mM
LG
siGAPDH
siGAPDH
siTRPM2
gp91phox
p22phox
p-PKC-α
PKC-α
P-ERK1/2
ERK1/2
β-actin
α/β Tubulin
gp91phox/β-actin
p22phox/β-actin
p-PKC-α/ total PKC-α
p-ERK1/2/total ERK1/2
LG+GA si
GA si
TRPM2 si
HG: 30mM
D
HG: 30mM
LG
-
3-AB
DPQ
p-p47phox
p47phox
TXNIP
GAPDH
p-p47phox / total p47phox
TXNIP/GAPDH
HG: 30mM

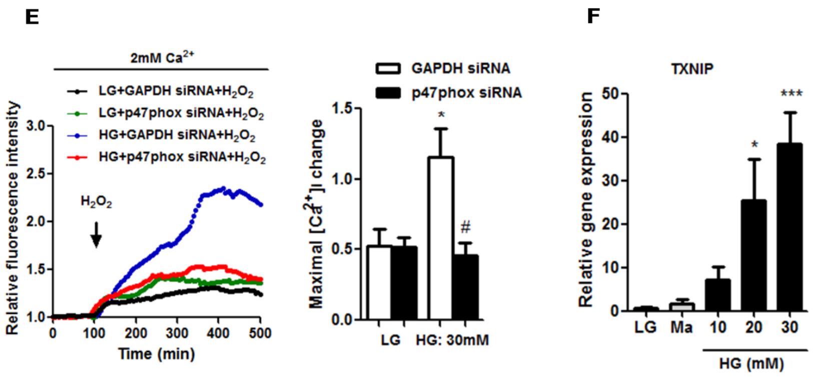

Supplementary Figure S3. (A) Representative immunoblots and graphs for protein expressions of p47phox, p22phox, TXNIP or GAPDH under low glucose (LG; 5.5mM glucose), mannitol (Ma; 30mM) or high glucose (HG; 10, 20, 30mM glucose) stimulation in U937 cells (n=4). (B) Graph for protein expressions of p22phox, gp91pox or GAPDH in the presence of BAPTA-AM (2.5, 5, 10μM), or EGTA (0.5, 1, 5mM) under LG or HG (n=4-5). (C) Representative immunoblots and graphs for protein expressions of gp91pox, p22phox, PKC-α, ERK1/2, β-actin or α/β-Tubulin in the presence of GAPDH- or TRPM2-siRNA under HG in U937 cells (n=4). (D) Representative immunoblots and graphs for protein expressions of p47phox, TXNIP or GAPDH in the presence of pre-treatment with 3-aminobenzamide (3-AB; 5mM) or 3,4-dihydro-5-[4-(1-piperidinyl)butoxy]-1(2H)-isoquinolinone (DPQ; 100μM) under HG (n=4). (E) Relative changes in intracellular Ca^{2+} concentration ($[Ca^{2+}]_i$) over the time course. U937 cells were treated in the presence of GAPDH- or p47phox-siRNA evoked by H_2O_2 (1mM) under LG or HG (n=4). (F) Quantitative PCR was performed on TXNIP mRNA, and it was normalized to LG. U937 cells were treated with LG, Ma

or HG for 48h (n=4). Data were shown as mean ± S.E.M. (A,B,D,F) *$P<0.05$*, **$P<0.01$ and ***$P<0.001$ vs. LG; $^{\#}P<0.05$ and $^{\#\#}P<0.01$vs. HG. (C,E) *$P<0.05$ and **$P<0.01$ vs. LG+GAPDH-siRNA; $^{\#}P<0.05$ vs. HG+GAPDH-siRNA.

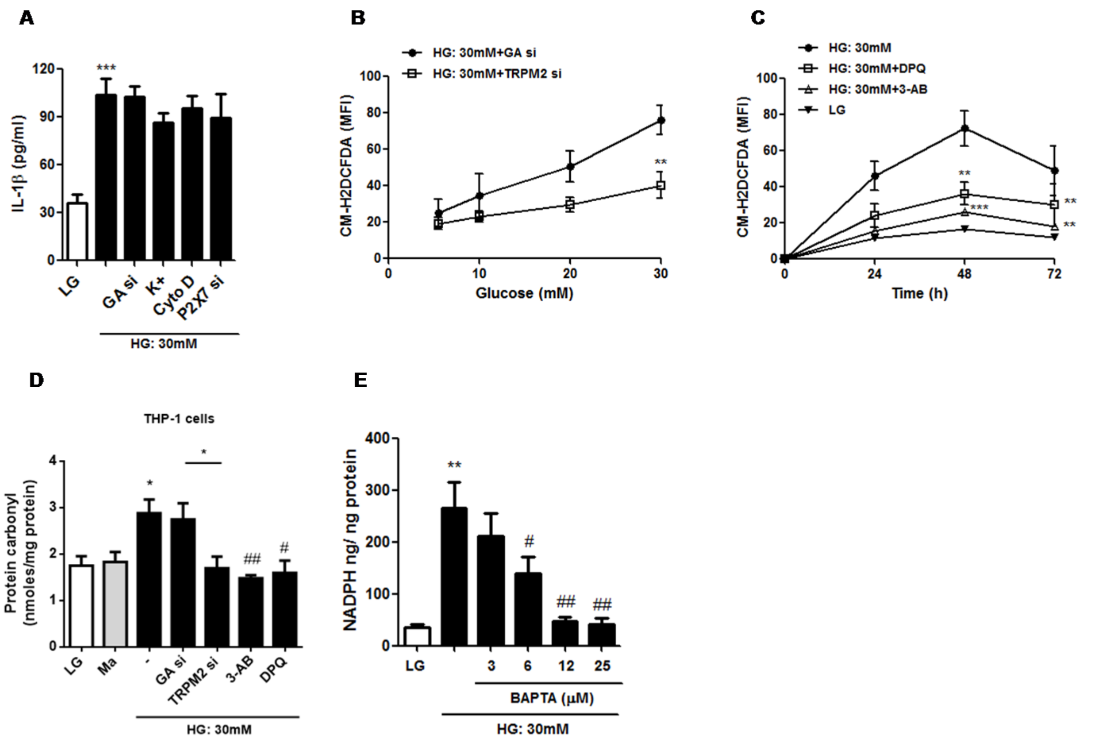

Supplementary Figure S4. (A) ELISA for IL-1β secretion from the supernatants of treated cells. (B,C) The ROS production was measured by CM-F2DCFDA. (D) The level of protein carbonyl content was measured by protein carbonyl content assay. (E) The cellular NADPH level was measured by NADP/NADPH assay, and NADPH oxidase activity was normalized to total cellular protein levels. (A,E) U937 cells were stimulated with low glucose (LG; 5.5mM glucose) or high glucose (HG; 30mM glucose) in the presence of (A) high K^+(60mM), cytochalasin D (cyto D; 2μM), GADPH- (GA si) or P2X7-siRNA (n=5), or (E) BAPTA-AM (3, 6, 12, 25μM) (n=4).

(B) U937 cells were stimulated with HG (10, 20, 30mM glucose) in the presence of GAPDH- or TRPM2-siRNA (n=4). (C) U937 cells were stimulated with HG for 24, 48 or 72h with pre-treatment of 3-aminobenzamide (3-AB; 5mM) or 3,4-dihydro-5-[4-(1-piperidinyl)butoxy]-1(2H)-isoquinolinone (DPQ; 100μM) (n=7). (D) THP-1 cells were pre-treated with 3-AB (5mM), DPQ (100μM), GAPDH- or TRPM2-siRNA under LG, mannitol (Ma; 30mM) or HG conditions (n=5). Data were shown as mean ± S.E.M. (A,D,E) $^{**}P<0.01$ and $^{***}P<0.001$ vs. LG; $^{\#}P<0.05$ and $^{\#\#}P<0.01$ vs. HG. (B) $^{**}P<0.01$ vs. HG+GAPDH-siRNA. (C) $^{**}P<0.01$ and $^{***}P<0.001$ vs. HG.
